# Pain suppresses corticospinal excitability, independent of tactile afferent inhibition

**DOI:** 10.1093/cercor/bhag041

**Published:** 2026-04-16

**Authors:** Louisa Gwynne, Luigi Tamè

**Affiliations:** School of Psychology, Keynes College, University of Kent, Canterbury, CT2 7NP, United Kingdom; School of Psychology, Keynes College, University of Kent, Canterbury, CT2 7NP, United Kingdom

**Keywords:** afferent inhibition, corticospinal excitability, pain, sensorimotor interaction

## Abstract

Pain can profoundly impact motor functioning to support self-preservation, yet its influence on the interaction between tactile input and corticospinal excitability (CSE) remains unclear. Across two experiments, a short- and long-latency afferent inhibition (AI) paradigm examined (i) whether tactile AI is modulated in the presence of tonic pain and (ii) the effect of pain on CSE in the presence of tactile afferent stimulation. In experiment 1, a single electrotactile stimulus (0.2- or 0.4-ms duration) was delivered to the index finger at one of six intervals (15 to 160 ms) before transcranial magnetic stimulation (TMS) over the ipsilateral first dorsal interosseous (FDI) hotspot. In experiment 2, the same procedure was tested during moderate, tonic forearm heat pain. Both experiments showed significant AI at 25, 35, and 160 ms delays, with facilitation at 60 ms. This effect was not influenced by the duration of afferent stimulation (experiment 1) nor by tonic heat pain (experiment 2). However, CSE was significantly reduced in painful compared to painless conditions (*P* = 0.021, η^2^_p_ = 0.132), indicating that while tonic pain modulates CSE, tactile afferent inhibition is unaffected. These findings show an inhibitory effect of pain on motor output that, in this context, occurs alongside preserved tactile-motor interactions.

## Introduction

Dynamic interaction between the somatosensory and motor systems permits environmental engagement, supporting adaptive responses and object manipulation (Johansson and Flanagan 2009; Zafarana et al. 2024). Somatosensory signaling, including nociceptive signals, can alter the functioning of the motor system and has been associated with disrupted corticospinal motor networks (Rohel et al. 2021) and sensorimotor disturbances (Harris 1999; McCabe et al. 2005). For example, hypertonic saline-induced muscle pain alters cortical motor circuits, reducing intracortical facilitation during pain and increasing inhibition postpain, as shown by changes in the transcranial magnetic stimulation (TMS)–evoked corticospinal excitability (CSE) (Schabrun and Hodges 2012). However, the relationship between cortical motor and somatosensory processes in pain remains unclear.

The modulatory effects of pain on corticospinal inhibitory and excitatory networks are well explored. Experimentally induced cutaneous thermal and muscle pain are largely associated with CSE inhibition (see Rohel et al. 2021; Chowdhury et al. 2022). Inhibition of TMS-induced CSE has been observed with both momentary (phasic) and continuous (tonic) thermal pain (Farina et al. 2001; Cheong et al. 2003; Dubé and Mercier 2011; Mavromatis et al. 2016; Billot et al. 2018), as well as tonic muscle pain (Schabrun and Hodges 2012; Burns et al. 2016a, 2016b; Larsen et al. 2018, 2019; Rice et al. 2021). However, findings are inconsistent (Rohel et al. 2021). For instance, neither hypertonic saline nor topical capsaicin-induced cheek pain affected CSE (Romaniello et al. 2000). Similarly, hypertonic saline-induced hand pain reduced excitability, whereas forearm pain had no effect (Larsen et al. 2018). Furthermore, CO_2_ laser pain delivered to the hand, but not the forearm, reduced CSE in the biceps brachii (Valeriani et al. 2001). Additionally, while one study found that topical capsaicin-induced tonic heat pain of the hand dorsum did not affect CSE of the ipsilateral FDI muscle (Mavromatis et al. 2016), another study reported that the effect of forearm pain on motor excitability was variable from one participant to another (Martel et al. 2017).

The motor and somatosensory cortices are interconnected, with primary somatosensory cortex (S1) projections to the primary motor cortex (M1), being predominantly inhibitory (Widener and Cheney 1997). This relationship is reflected in the modulation of TMS-induced motor-evoked potentials (MEPs) by tactile stimulation (Chen et al. 1999; Tokimura et al. 2000; Tamè and Holmes 2023). A single electrocutaneous stimulus can inhibit CSE (Chen et al. 1999; Tokimura et al. 2000; Tamburin et al. 2005;Bailey et al. 2016 ; Turco et al. 2017), depending on the delay between afferent input and TMS delivery. Inhibition occurs at short (<40 ms) or long delays (≥80 ms), termed short- and long-latency afferent inhibition (SAI and LAI, respectively; Chen et al. 1999; Tokimura et al. 2000). Moreover, the magnitude of afferent inhibition increases with greater stimulation intensities (Bailey et al. 2016; Turco et al. 2017).

Nociceptive processing can modulate afferent inhibition (AI). For example, SAI and LAI in the right first dorsal interosseous (FDI) muscle were reduced after, but not during, saline-induced muscle pain (Burns et al. 2016b). Conversely, tonic cold-induced pain reduced SAI both during and immediately after pain, with no effect on LAI (Delahunty et al. 2024). In a different context, acute cutaneous heat pain had no impact on SAI when transient painful stimulation was applied simultaneously with the afferent stimulation (Mercier et al. 2016).

Overall, the effects of nociceptive pain on such tactile sensory and motor interactions remain unclear, with limited research preventing a comprehensive understanding. Assessing the effects of pain on CSE, and, in particular, the nature of such effects during ongoing tactile-CSE interactions as indexed by AI, offers a valuable framework to understand how nociceptive inputs influence motor output and to unravel any relationship between tactile and painful inputs that mediate CSE. These insights have clinical relevance for understanding motor alterations and atypical sensory and motor functioning in pain-related disorders. Notably, SAI has been proposed as a research tool and potential biomarker for neurological pathologies including Parkinson’s and Alzheimer’s disease (Di Lazzaro et al. 2002; Sailer et al. 2003; Yarnall et al. 2013; Choudhury et al. 2023).

The objectives of the current study were 2-fold. First, experiment 1 aimed to characterize and optimize an AI paradigm in order to (i) validate the temporal profile by which brief tactile stimulation modulates CSE and (ii) determine whether the duration of afferent stimulus (0.4 vs. 0.2 ms) alters the magnitude of AI. In our paradigm, a single transcutaneous electrical pulse was delivered to the left index finger preceding single-pulse TMS (spTMS) over the contralateral motor cortex at one of six interstimulus intervals (15, 25, 35, 45, 60, or 160 ms). Although previous studies have shown that perceivably stronger intensities of afferent stimulation can increase AI (Bailey et al. 2016), it remains unknown whether a longer pulse duration, which can recruit additional tactile afferents without altering perceived intensity, produces similar effects. We hypothesized significant inhibition of CSE at short (25 to 35 ms) and long (>100 ms) delays, consistent with prior literature (eg Turco et al. 2018), and greater inhibition for the longer afferent duration. This expectation was based on findings that longer electrical pulses progressively recruit a larger population of afferent fibers, enhancing the immediate sensory volley, but do not themselves produce a prolonged baseline shift in CSE beyond transient momentary effects (Chipchase et al. 2011).

Experiment 2 aimed to determine: (i) whether tonic cutaneous heat pain modulates tactile AI and (ii) whether any effect of tonic pain on CSE differs depending on the presence and timing of tactile input. This approach allowed for the assessment of both the influence of pain on AI and the extent to which pain-related shifts in CSE may vary across the temporal structure of tactile-motor interactions. While prior works have shown that experimentally induced hand pain can suppress CSE of ipsilateral limb muscles (Mercier et al. 2016; Martel et al. 2017; Delahunty et al. 2024), findings into the effects of forearm pain are inconsistently reported (Valeriani et al. 2001; Martel et al. 2017; Larsen et al. 2018). Moreover, only a small number of studies have evaluated whether pain modulates tactile-evoked AI and, as of yet, no study has investigated this using a model of tonic heat pain, nor do they investigate if the variable effects of pain on CSE may be influenced by the presence of simultaneous tactile afferent-motor interactions spanning a temporal window capable of assessing SAI, LAI, and recovery. In experiment 2, using the optimized design from experiment 1, tonic moderate heat pain was applied to the left forearm while participants completed the same AI protocol. It was hypothesized that tonic pain would alter tactile AI, whether attenuating, enhancing, or leaving it unchanged, given the mixed and limited findings in the existing literature. It was also hypothesized that tonic pain may overall alter CSE, consistent with previous reports on the effects of nociceptive input on CSE; however, this effect may be mediated by simultaneous transient afferent stimulation (Farina et al. 2003).

## EXPERIMENT 1: Corticospinal excitability as a function of time and afferent duration

### Materials and methods

#### Participants

Twenty participants were recruited *(Mean_Age_* = 20.5, *SD_Age_* = 5.6 years; 6 males, 14 females). All participants were right-handed as indicated by self-report on the Edinburgh Handedness Inventory questionnaire (EHI; Oldfield 1971; *M* = 85, *range* = 33 to 67). Participants had no neurological, pain-related, or psychiatric conditions and no current use of psychiatric and neuroactive medications or consumption of analgesic substances at least 24 h before participation. All participants provided informed consent before testing, and all procedures were approved by the School of Psychology, University of Kent ethics committee (Ethics ID: 202417277059689279) and adhered to published TMS safety guidelines (Rossi et al. 2021). A priori power analysis in G^*^Power (version 3.1.9.7; Faul et al. 2007) indicated that a sample of 20 participants would provide power (80%) to detect a minimum effect size (η^2^_p_) of 0.060 (α = 0.05) for the within-subjects interaction (Delay × Stimulus Length).

#### TMS and neuronavigation

TMS procedures are reported in line with guidelines recommended for studies investigating the motor system (Chipchase et al. 2012). Biphasic spTMS was delivered with a MAG and More PowerMAG 100 Repetitive Magnetic Stimulator via a 70 mm-diameter figure-of-eight coil. Electromyography (EMG) was recorded with two Ag-AgCI surface electrodes placed over the first dorsal interosseus (FDI) muscle of the left hand in a belly-tendon montage with ~2 cm interelectrode distance. The ground electrode was placed 1 to 2 cm proximal to the left pisiform bone of the left wrist, and skin preparation was completed using an alcohol swab. The EMG signal was sampled in BrainSight Neuronavigation software (version 2.4.10; Rogue Research Inc., Montreal, QC) with a 3,000 Hz sampling frequency. Scalp localization and coil orientation were optimized using the same BrainSight Neuronavigation, based on the functionally identified FDI motor hotspot and its corresponding Montreal Neurological Institute (MNI) stereotaxic coordinates.

TMS intensity for the main experimental protocol was set at 110% of the optimal scalp coil positioning and TMS intensity to elicit a 50 μV peak-to-peak MEP in the left FDI muscle while at rest (resting motor threshold; RMT) in at least 5 out of 10 trials. The mean right M1-FDI RMT was 43.1% (*SD* = 5.53; Table 1). A detailed description of M1-FDI thresholding can be found in Supplementary Materials S1.

**Table 1:** TMS thresholds (%) and electrotactile sensory detection thresholds (SDT; mA) across experiments and tactile conditions.

**Participant**	**Experiment 1**				**Experiment 2**	
	**Session 1**		**Session 2**			
	**MSO**	**SDT**	**MSO**	**SDT**	**MSO**	**SDT**
1	44.0	1.37	44.5	1.69	38.0	1.86
2	39.0	0.77	41.0	1.17	36.5	1.25
3	39.5	0.87	40.0	1.69	38.5	0.92
4	43.0	0.95	45.5	1.73	51.5	1.42
5	43.5	0.57	43.0	0.85	43.0	1.10
6	50.5	0.73	51.5	1.01	50.0	0.97
7	35.5	0.84	35.5	1.39	41.0	0.89
8	43.0	1.29	41.5	1.77	57.0	1.44
9	47.5	1.07	48.0	1.93	40.0	1.71
10	38.5	1.15	39.0	1.81	52.0	1.23
11	39.5	0.99	40.0	1.69	42.5	1.89
12	45.0	1.02	47.0	1.64	49.0	1.61
13	40.5	1.09	40.5	1.33	44.5	2.04
14	43.0	0.84	39.5	1.45	49.0	1.11
15	40.5	1.20	41.0	2.03	44.5	1.51
16	48.0	0.73	46.5	1.34	29.0	1.20
17	41.0	0.97	40.5	1.15	51.0	2.68
18	40.0	0.88	40.0	1.41	37.5	1.33
19	34.5	1.29	34.5	1.77	47.5	0.89
20	48.5	1.11	48.5	1.65	34.0	1.29
**Mean** **SD**	42.2 4.22	0.99 0.21	42.3 4.37	1.52 0.31	43.8 7.09	1.42 0.45

Note. MSO, maximum stimulator output (%) of the resting motor threshold (RMT); SD, standard deviation.

#### Electrocutaneous stimulation

Innocuous transcutaneous electrical stimulation was delivered by a bipolar constant current stimulator (DS5; Digitimer, Welwyn Garden City, United Kingdom) through two stainless-steel electrode rings fitted at the proximal and intermediate phalanges of the participants’ left index finger. Square-wave pulse widths were either 0.4 or 0.2 ms (sessions 1 and 2, respectively) and were delivered at 2.5 times the participants’ sensory detection threshold (SDT). SDT estimation procedures can be found in Supplementary Materials S2. For both the 0.2- and 0.4-ms tactile stimulation conditions, the experimental stimulation level was always calculated relative to the specific SDT of that pulse duration. SDTs (mA) were significantly lower for the 0.4-ms pulse than the 0.2-ms pulse (*t* = 11.67, *P* < 0.001; Table 1), reflecting the fact that longer pulse durations require less current to produce the same perceptual intensity. As previous studies have shown that perceivably stronger intensities can induce greater SAI (Bailey et al. 2016), scaling stimulation intensity relative to the SDT for each duration allowed us to vary pulse duration while keeping perceived subjective intensity comparable between the 0.2- and 0.4-ms conditions.

#### Experiment protocol

**Figure 1:**
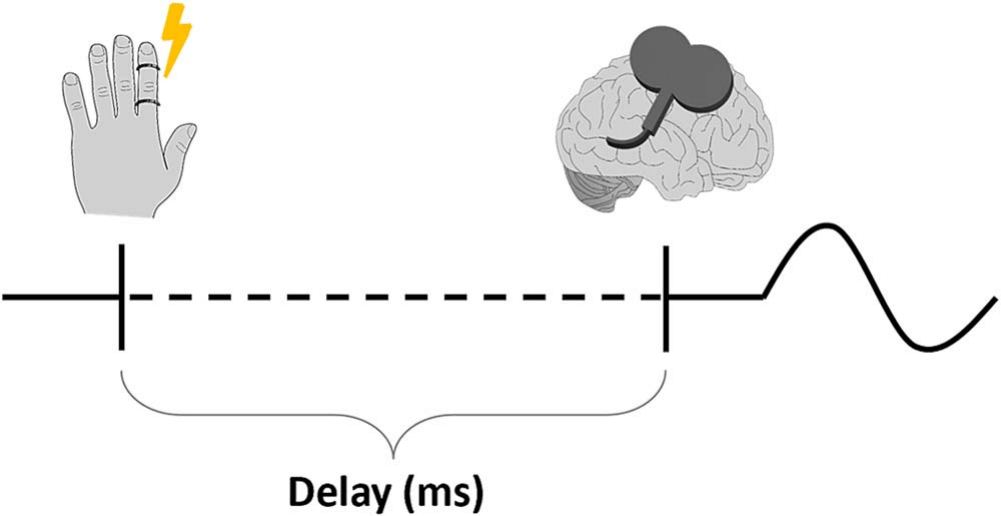
Schematic depiction of the experiment trials. An electrocutaneous stimulus was delivered to the left index finger through two ring electrodes. After afferent stimulation onset, spTMS over right-M1 was delivered at a single delay of 15, 25, 35, 45, 60, or 160 ms following a random order.

Participants sat at a desk with their left arm visibly resting naturally in front of them. They were instructed to fixate on a central black cross on a gray screen ~60 cm in front of them. The protocol comprised 8 blocks of 35 trials with a 5-s intertrial interval. From the onset of every trial, an electrotactile stimulus was delivered at a randomly jittered onset (0 to 1 s), followed by spTMS (Fig. 1). spTMS occurred at one of six temporal delays (15, 25, 35, 45, 60, or 160 ms) after tactile onset. Early delays (<100 ms) were selected, given that S1 activity persists at least 60 ms after tactile offset (Allison et al. 1992; Mauguière et al. 1997), while the 160-/ms delay reflected long-latency AI (Hamada et al. 2002). Control trials (TMS-only) included a fixed 40-ms spTMS delay from the hypothetical onset of the electrotactile stimulus (0 mA). Each block comprised five trials per delay and five control trials, randomized in order.

#### Design

Sessions 1 and 2 were completed under a within-participants design and conducted at least 48 h apart (days between sessions: *Mean* = 7.75, *Range* = 2 to 17). MEP amplitudes were calculated as the peak-to-peak amplitude in the 10- to 90-ms time window after the TMS pulse. To quantify AI, that is, the change in CSE by a preceding tactile stimulus, MEP amplitudes at each conditioned delay were averaged across trials and blocks and then normalized to the TMS-only (unconditioned) mean amplitude. This yielded an MEP ratio reflecting the proportion of inhibition or excitation of the motor response due to afferent input. MEP ratios were therefore computed as: *MEP ratio = (conditioned MEP amplitude)/(TMS-only MEP amplitude)*. To improve data distribution, MEP ratios were then log-transformed, meaning that an MEP ratio of 0 (*SD* = 0) corresponds to the TMS-only baseline. To firstly assess for AI, MEP ratios at each delay (15, 25, 35, 45, or 60 ms) were compared against zero (TMS-only baseline) using false discovery rate (FDR)–corrected two-tailed *t*-tests, with an MEP ratio significantly less than 0, indicating significant inhibition. Following this initial assessment of AI, to test the effect of afferent duration on AI, a two-way repeated-measures analysis of variance (RM ANOVA) was conducted on the within-participant factors DELAY, examined on five levels (15, 25, 35, 45, and 60 ms), and AFFERENT DURATION, examined on two levels (0.4 vs. 0.2 ms). Post hoc pairwise comparisons were performed with a Tukey’s honestly significant difference (HSD) correction.

As delays of ≥100 ms are considered to reflect LAI, the 160-ms delay was analyzed separately from the main ANOVA. A paired-samples *t*-test between MEP ratios at 0.2- and 0.4-ms afferent durations assessed whether LAI differed between the two conditions.

## Results

FDR-corrected two-tailed *t*-tests comparing MEP ratios at each delay to 0 (TMS-only trials) showed significant inhibition for both 0.2- and 0.4-ms afferent durations at delays of 25 ms (0.2: *M* = −0.17, *SE* = 0.04; *t*(19) = −4.75, *P_FDR_* = 0.002, *d* = −1.06; 0.4: *M* = −0.24, *SE* = 0.03; *t*(19) = −7.15, *P_FDR_* = 0.002, *d* = −1.60), 35 ms (0.2: *M* = −0.09, *SE* = 0.03; *t*(19) = −3.26, *P_FDR_* = 0.008, *d* = −0.73; 0.4: *M* = −0.18, *SE* = 0.04; *t*(19) = −4.33, *P_FDR_* = 0.002, *d* = −0.97) and 160 ms (0.2: *M* = −0.35, *SE* = 0.05; *t*(19) = −7.00, *P_FDR_* = 0.002, *d* = −1.57; 0.4: *M* = −0.37, *SE* = 0.04; *t*(19) = −9.24, *P_FDR_* = 0.002, *d* = −2.07). At a 60-ms delay, MEP ratios were significantly greater than 0 with a 0.4-ms afferent duration (0.4: *M* = 0.13, *SE* = 0.05; *t*(19) = 2.68, *P_FDR_* = 0.026, *d* = 0.6), indicating facilitation, but not with a 0.2-ms duration (0.2: *M* = 0.09, *SE* = 0.04; *t*(19) = 2.04, *P_FDR_* = 0.084, *d* = 0.46). No early significant inhibition was observed at 15 ms for either afferent durations (0.2: *M* = −0.00, *SE* = 0.03; *t*(19) = −0.11, *P_FDR_* = 0.916, *d* = −0.02; 0.4: *M* = −0.00, *SE* = 0.02; *t*(19) = −0.21, *P_FDR_* = 0.916, *d* = −0.05). Lastly, no inhibition was observed at 45 ms; MEP ratios did not differ from 0 for either duration (0.2: *M* = 0.02, *SE* = 0.04; *t*(19) = 0.48, *P_FDR_* = 0.763, *d* = 0.11; 0.4: *M* = 0.03, *SE* = 0.04; *t*(19) = −0.66, *P_FDR_* = 0.692, *d* = −0.15), consistent with recovery.

Mauchly’s test of the 2-way RM ANOVA testing the effect of afferent stimulus duration on AI (DELAY × STIMULUS LENGTH) revealed violation of sphericity for DELAY (*P* < 0.001), so Greenhouse–Geisser correction was applied. A main effect of DELAY was observed; *F*(2.13, 40.46) = 26.24, *P* < 0.001, η^2^_p_ = 0.580. Post hoc corrected comparisons evidenced that MEP ratios significantly differed across temporal delays. Specifically, MEP ratios at 25 ms were significantly lower than at 15 ms (*t*(19) = −5.14, *P_tukey_* < 0.001), 35 ms (*t*(19) = −3.30, *P_tukey_* = 0.027), 45 ms (*t*(19) = 5.46, *P_tukey_* = < 0.001), and 60 ms (*t*(19) = 7.10, *P_tukey_* < 0.001). Similarly, MEP ratios at a 35-ms delay were significantly lower than at 15-ms (*t*(19) = −5.14, *P_tukey_* < 0.001), 45 ms (*t*(19) = −3.97, *P_tukey_* = 0.006), and 60 ms (*t*(19) = −5.16, *P_tukey_* < 0.001). At 45 ms, MEP ratios did not significantly differ from 15 ms (*t*(19) = 0.06, *P_tukey_* = 0.100). In contrast, at a 60-ms delay, MEP ratios were significantly greater compared to 45 ms (*t*(19) = 3.89, *P_tukey_* = 0.008). These patterns collectively demonstrate the sequence of SAI (25 and 35 ms), recovery at 45 ms, and facilitation around 60 ms.

**Figure 2:**
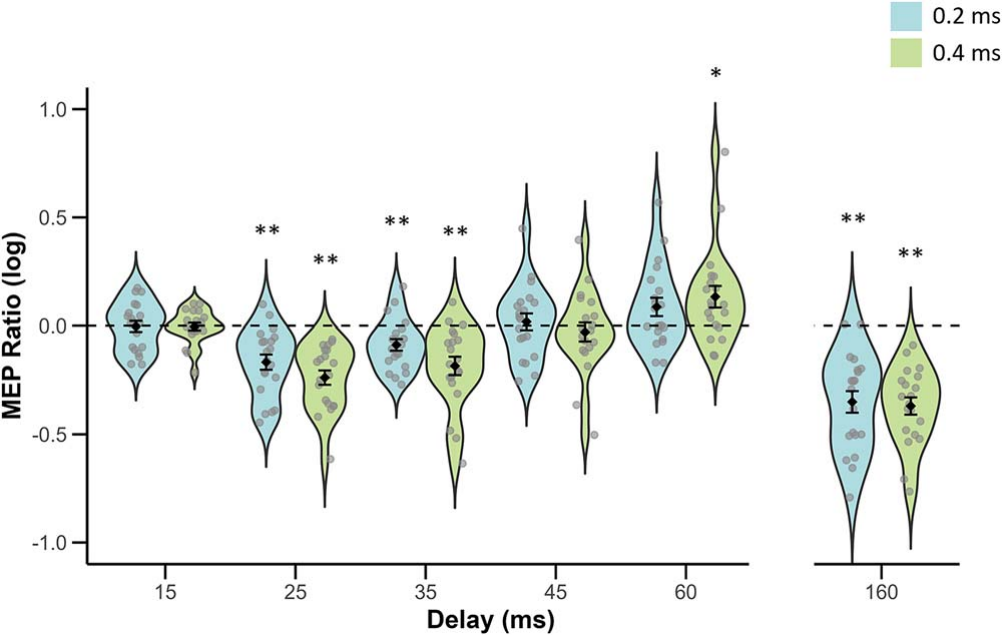
Violin plots show mean and standard error of the mean (SEM) MEP ratios as a function of afferent stimulus duration and delay (ms). An MEP ratio of 0 represents TMS-only trials and is represented by the dashed line. Gray circles show individual participant data points. Asterisks indicate delays (ms) in which MEP ratios significantly differed from 0 (unconditioned, TMS-only, MEP ratio) based on FDR corrected two-tailed *t*-tests. ^*^P < 0.05, ^**^P < 0.01, ^***^P < 0.001.

There was no significant main effect of STIMULUS LENGTH; *F*(1,19) = 1.22, *P* = 0.283, η^2^_p_ = 0.06. However, a significant DELAY × STIMULUS LENGTH interaction emerged; *F*(4, 76) = 3.00, *P* = 0.023, η^2^_p_ = 0.136. Despite the statistical significance of this interaction, post hoc comparisons did not reveal any delay at which MEP ratios differed significantly between 0.2- and 0.4-ms afferent durations after correction. Thus, the interaction does not reflect reliable duration-specific effects at individual delays but instead may arise from opposing non-significant trends at 25- and 60-ms delays between 0.2 and 0.4 ms stimuli (Fig. 2).

To assess any effect of afferent duration on LAI, a paired-samples *t*-test was conducted to compare MEP ratios at a delay of 160 ms. This revealed no significant difference in MEP ratios between the 0.2-ms (*M* = −0.35, *SE* = 0.05) and 0.4-ms (*M* = −0.37, *SE* = 0.04) afferent durations at a 160-ms delay; *t*(19) = 0.33, *P* = 0.747, *d* = 0.07. For completeness, the 2-way RM ANOVA was repeated including the 160-ms delay, though the results were unaltered.

## Discussion

In experiment 1, we found that a single electrocutaneous stimulus to the left index finger inhibited MEPs of the left FDI muscle at both early (ie 25- and 35-ms) and late (160-ms) delays. This finding is consistent with previous evidence of motor inhibition at short (Tokimura et al. 2000; Tamburin et al. 2001, 2005; Bikmullina et al. 2009; Udupa et al. 2009, 2014; Cash et al. 2015; Tamè et al. 2015; Bailey et al. 2016) and long (Chen et al. 1999; Sailer et al. 2002; Turco et al. 2017) interstimulus intervals. Contrary to our hypothesis, the magnitude of AI was not affected by the length of the afferent stimulus, suggesting that the modulation of AI by increased afferent recruitment is selective.

## EXPERIMENT 2: Corticospinal excitability and afferent inhibition as a function of tonic heat pain

Experiment 1 confirmed the validity of our approach and established the temporal profile by which transient tactile input modulates CSE. Building on this, experiment 2 used the same AI design to test whether tonic cutaneous heat pain alters AI and whether possible pain-related changes in CSE vary as a function of transient tactile input. Because experiment 1 showed that tactile input can suppress, facilitate, or leave CSE unchanged depending on its timing, experiment 2 assessed whether pain-related changes in CSE, or the absence of such change, differs across these temporally specific tactile–motor interactions.

## Materials and methods

### Participants

Twenty new participants were recruited *(Mean_Age_* = 20.5, *SD_Age_* = 5.69 years; 6 males, 14 females). Fifteen were right-handed, 3 left-handed, and 2 ambidextrous as recorded by the Edinburgh Handedness questionnaire (EHI; Oldfield 1971; *M* = 55, range = −100 to 100). Recruitment methods and exclusion criteria were identical to experiment 1.

### Experiment protocol

**Figure 3:**
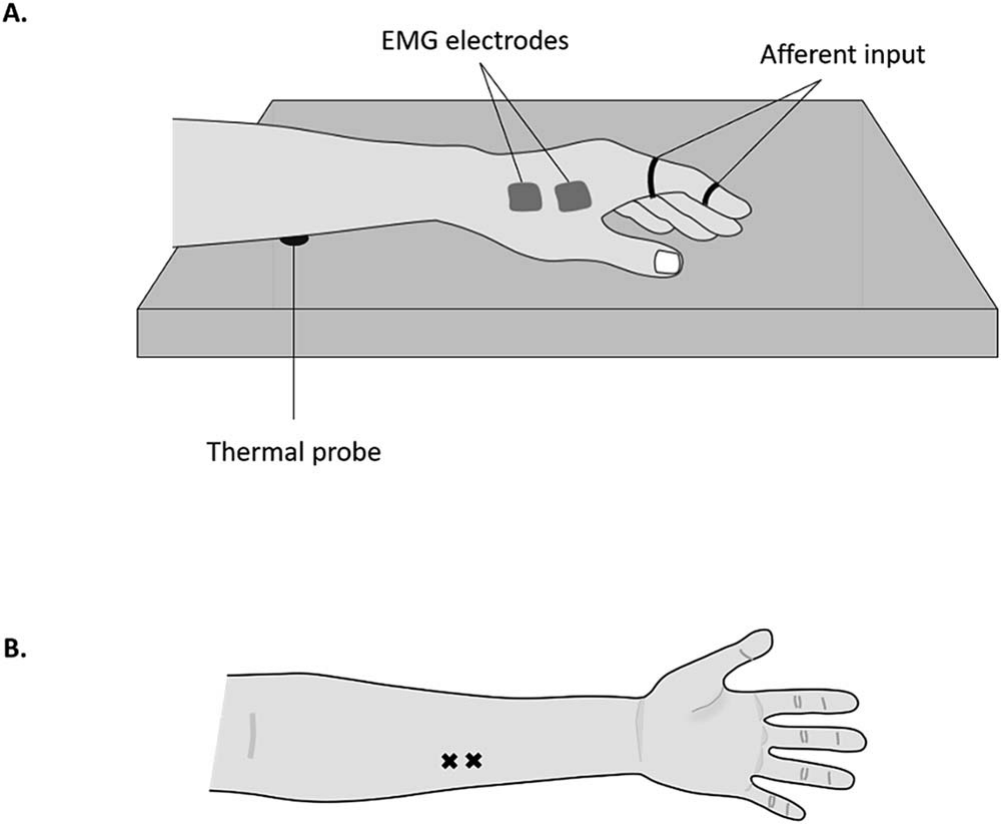
The experimental set-up. A) Illustration of the set-up of thermal heat pain induction; the thermode was integrated into the desk surface, and the arm remained rested on the probe throughout. B) Two points of thermode contact were marked on the right forearm.

The experimental protocol was identical to experiment 1 with the following exception: Continuous moderate heat pain was applied to the left forearm throughout every block of trials. The thermode was integrated into the wooden desk with the probe tip flush with the tabletop (Fig. 3A). This allowed for thermal stimulation to be delivered without additional tactile input elsewhere on the body.

### Thermal stimulation

After TMS and electrotactile thresholding were performed, a moderate level of heat pain was calibrated. Cutaneous tonic heat pain was induced on the participants’ left forearm by a 13 mm-diameter contact thermal probe (NTE-2A Thermal Probe and controller; Physitemp Instruments LLC, Clifton, NJ, USA) along with topical application of a 0.2% capsaicin cream. The topical application of low-concentration capsaicin cream allows for sensitization of C-nociceptive fibers for mild heat (<40°C) to be perceived as painful while minimizing the risk of tissue damage (Baumann et al. 1991). Two thermode stimulation points were marked on the participant’s arm, one midway between the center right of the cubital fossa and the wrist joint, and the second 2 cm distal to the first point (Fig. 3B). These markers served as locations of thermal stimulation to avoid potential skin damage from prolonged thermode contact and to minimize habituation or sensitization over time. A half-pea-sized amount of capsaicin was applied to each marker for 10 min, then removed with a dry cloth before the start of the main experimental protocol. Five participants reported a mild but not painful warm and/or tingling sensation that resolved before thermode stimulation was added. All other participants reported no perceptible sensations. Painful heat stimulation for the experimental protocol was calibrated to elicit a verbal rating of 7 on a pain scale (0 = “no pain at all,” 10 = “worst pain imaginable in the current context”). Starting at the baseline temperature (35°C), the temperature was manually increased in 0.5°C increments until the desired heat rating was attained. The thermode was moved between two sites of thermal stimulation at the start of each block under an ABAB counterbalance design and the thermode temperature was adjusted in increments of 0.5°C to maintain a moderate level of pain (7/10). Thermode temperature required to elicit moderate pain significantly increased across blocks as indicated by a nonparametric Friedman test; *X^2^* (7) = 27.08, *P* = 0.001. The mean temperature delivered to elicit moderate pain (7/10) was 40.6°C (*SD =* 3.6°C).

### Design

Given that experiment 1 showed afferent durations of 0.2 and 0.4 ms had no effect on AI and in accordance with previous reports, the electrotactile pulse width was set at 0.2 ms (Chen et al. 1999; Sailer et al. 2002). The control (painless) group for comparison with the painful group of experiment 2 was taken from experiment 1, session 2, which employed the same 0.2-ms afferent stimulation duration and otherwise identical AI procedure. These painless data were used in all between-participant analyses comparing painless and painful states (ie PAIN STATE (painless vs. painful) including both the MEP ratios and raw untransformed MEP amplitudes.

AI was quantified using the same procedures as experiment 1. For each delay, MEP amplitudes were extracted as peak-to-peak amplitudes and converted to MEP ratios by normalizing conditioned MEPs to the mean amplitude of TMS-only trials (unconditioned baseline). Ratios were log-transformed to improve distributional properties, such that a value of 0 corresponds to the TMS-only baseline. To assess the presence of AI at each delay, MEP ratios were first compared to 0 using two-tailed on-sample *t*-tests with an FDR correction for multiple comparisons. To test whether tonic pain modulated AI, MEP ratios were analyzed with a two-way mixed-effects ANOVA on the within-participant factor DELAY (15, 25, 35, 45, or 60 ms) and between-participants factor PAIN STATE (painless vs. painful). Responses at a 160 ms delay were analyzed separately to assess the effect of pain on LAI using an independent-samples *t*-test comparing MEP ratios between PAIN STATE groups.

To further assess the effect of tonic pain on CSE and, whether this effect differs depending on the presence and timing of tactile afferent input, raw (untransformed) MEP amplitudes were analyzed using a second two-way mixed effects ANOVA with the between-participants factor PAIN STATE (painless vs. painful) and within-participant factor AFFERENT STIMULATION (None, 15, 25, 35, 45, 60, or 160 ms). This analysis tested whether any effect of pain on CSE varies across conditions of tactile stimulation, which itself can modulate CSE (as established in experiment 1).

## Results

**Figure 4:**
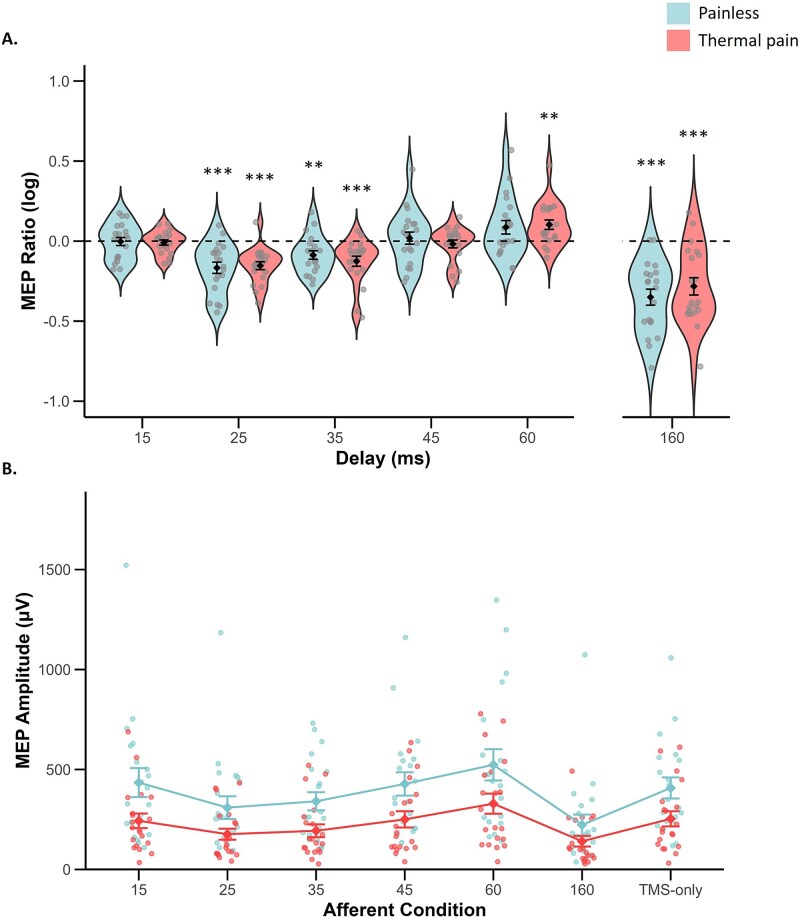
A) Violin plots show mean and standard error of the mean (SEM) MEP ratios as a function of pain state and delay (ms). An MEP ratio of 0 represents TMS-only trials and is represented by the dashed line. Circles show individual participant data points. B) shows mean and SEM MEP amplitudes as a function of pain state across transient types of tactile afferent stimulation. Faded colored circles are individual participant data points. Asterisks indicate delays (ms) in which MEP ratios significantly differed from 0 (unconditioned, TMS-only, MEP ratio) based on FDR corrected two-tailed *t*-tests. ^*^P < 0.05, ^**^P < 0.01, ^***^P < 0.001.

FDR corrected two-tailed *t*-tests comparing MEP ratios at each delay to 0 (TMS-only trials) for the painful group (experiment 2) showed significant inhibition at 25 ms (*M* = −0.15, *SE* = 0.03; *t*(19) = −6.23, *P_FDR_* = 0.002, *d* = −1.39), 35 ms (*M* = −0.13, *SE* = 0.03; *t*(19) = −3.99, *P_FDR_* = 0.002, *d* = −0.89) and 160 ms (*M* = −0.28, *SE* = 0.05; *t*(19) = 5.22, *P_FDR_* =. 0.002, *d* = −1.17). At 60 ms, MEP ratios were significantly greater than 0 (*M* = 0.10, *SE* = 0.03; *t*(19) = 3.49, *P_FDR_* = 0.003, *d* = 0.78. MEP ratios did not differ from 0 at delays of 15 ms (*M* = −0.01, *SE* = 0.02; *t*(19) = −0.54, *P_FDR_* = 0.595, *d* = −0.12) or 45 ms (*M* = −0.02, *SE* = 0.02; *t*(19) = −0.73, *P_FDR_* = 0.476, *d* = 0.78). Mauchly’s test of the 2-way mixed-effects ANOVA testing the effect of pain on AI (DELAY x PAIN STATE) revealed violation of sphericity for DELAY (*P* < 0.001), so a Greenhouse–Geisser correction was applied. This revealed a main effect of DELAY, *F*(2.85, 108.33) = 31.24, *P* < 0.001, η^2^_p_ = 0.451, no main effect of PAIN STATE, *F*(1,38) = 0.132, *P* = 0.719, η^2^_p_ = 0.003, and no interaction (DELAY × PAIN STATE), *F*(4, 152) = 0.54, *P* = 0.706, η^2^_p_ = 0.014 (Fig. 4A). An independent two-tailed *t*-test comparing MEP ratios at a 160-ms delay revealed no significant difference between the painful (*M* = −0.28, *SE* = 0.05) and painless (*M* = −0.35, *SE* = 0.05) conditions; *t*(38) = 0.92, *P* = 0.365, *d* = 0.3.

Mauchly’s test of the 2-way mixed-effects ANOVA testing the effect of pain on CSE across differing tactile afferent input revealed violation of sphericity for AFFERENT STIMULATION (*P* < 0.001), so a Greenhouse–Geisser correction was applied. There was a main effect of AFFERENT STIMULATION, *F*(2.87, 108.85) = 24.57, *P* < 0.001, η^2^_p_ = 0.393, reflecting inhibition and facilitation in MEP amplitudes before ratio transformations. While this did not vary as a function of pain AFFERENT STIMULATION × PAIN STATE, *F*(6, 228) = 1.50, *P* = 0.180, η^2^_p_ = 0.038), there was a significant main effect of PAIN STATE, *F*(1,38) = 5.78, *P* = 0.021, η^2^_p_ = 0.132. As shown in Fig. 4B, MEP amplitudes were significantly lower in the painful (*M* = 226.64, *SE* = 45.34) than painless group (*M* = 380.81, *SE* = 45.34; *t*(38) = −2.40, *P* = 0.021).

As a control, tactile detection thresholds and TMS M1-FDI motor thresholds were compared between the painful and painless conditions. Two-tailed independent-samples *t*-tests showed no significant differences in tactile detection thresholds (painful: *M* = 1.42 mA, *SE* = 0.1; painless: *M* = 1.53 mA, *SE* = 0.07; *t*(38) = −0.88, *P* = 0.385, *d* = −0.28) or TMS motor thresholds (painful: *M* = 43.8%, SE = 1.59; painless: *M* = 42.4%, SE = 0.98; *t*(38) = 0.77, *P* = 0.449, *d* = 0.24).

## Discussion

Experiment 2 found that moderate tonic heat pain applied to the forearm produced a general suppression of CSE in the ipsilateral FDI muscle. This was indicated by significantly reduced MEP amplitudes across all afferent stimulation conditions. Importantly, despite this overall reduction in excitability, tactile AI was preserved in that pain did not significantly alter AI, and the temporal pattern of inhibition and recovery was comparable between the painful and painless groups. Thus, the present results show that tonic nociceptive pain reduces CSE, but this reduction occurs in parallel with intact tactile–CSE interactions.

Our findings are consistent with those of Burns et al. (2016b) who reported no effect of saline-induced muscle pain in the tested muscle on SAI (Burns et al. 2016b). However, the present study extends this finding to cutaneous thermal pain applied to the forearm proximal to both the tactile input and the tested muscle. Additionally, we broaden the scope of this finding across temporal delays. While Burns et al. (2016b) examined only 20-ms (SAI) and 200-ms (LAI) interstimulus intervals, we demonstrated that the preservation of AI during tonic pain also applies to the afferent facilitation observed at a 60-ms delay and to motor recovery at 45 ms.

## General discussion

In the present study, we used an AI paradigm to investigate the effects of tonic pain on CSE and transient tactile–motor interactions. Cutaneous heat pain applied to the forearm significantly reduced MEP amplitudes of the ipsilateral FDI muscle, signifying suppressed motor output. Despite this reduction, neither SAI nor LAI was affected by pain, consistent with some previous studies (Mercier et al. 2016; Burns et al. 2016b) but contrasting others such as Delahunty et al. (2024), who reported reduced SAI during painful cold-water immersion of the contralateral hand (Delahunty et al. 2024). This discrepancy may be related to differences in the nature and physiological consequences of pain stimuli used across studies. For example, cold-water immersion paradigms introduce a broader alteration to the somatosensory environment, incorporating stronger and less localized cutaneous and thermoreceptive inputs, making it difficult to isolate the effect of nociception from other sensory drivers of CSE. Moreover, intramuscular pain not only activates nociceptors but also induces local metabolic and inflammatory processes, which may modify excitability within the same corticospinal pathway being tested. Resultingly, any observed change in AI may reflect combined nociceptive input and local muscle perturbation rather than pain per se. Instead, the current study employed a pain model with more isolated cutaneous nociceptive drive, avoiding local changes to muscle physiology or widespread somatosensory disruption. Additionally, we found no pain-related effects on afferent facilitation (60-ms interstimulus interval) or MEP recovery (45 ms), thereby extending previous findings. Collectively, our results indicate that while tonic pain suppresses CSE, tactile AI is undisturbed.

The finding that tonic heat pain exerts an inhibitory influence on motor output aligns with previous studies demonstrating reduced CSE in response to painful heat exposure (Farina et al. 2001; Cheong et al. 2003; Dubé and Mercier 2011; Mavromatis et al. 2016; Mercier et al. 2016; Billot et al. 2018). Notably, we emphasize this finding in light of a recent meta-analysis suggesting limited evidence to show the effect of tonic forearm pain on CSE (Rohel et al. 2021). One possible explanation for the suppression of CSE is that the downregulation of corticomotor activity facilitates the prioritization of adaptive spinal reflex responses (Inghilleri et al. 1997; Farina et al. 2003). Additionally, suppressed CSE may reflect central nervous system mechanisms aimed at minimizing further pain and tissue damage (Hodges and Tucker 2011).

Alternatively, we propose a role for executive control in maintaining limb and body posture against an ongoing noxious stimulus. In the current experiment, thermal stimulation was calibrated to elicit moderate pain (rated 7 out of 10 on the pain scale), and participants were instructed to remove the limb only if pain became intolerable-we note that this situation never occurred. The observed suppression of MEPs in the ipsilateral FDI may reflect the exertion of such top-down control in experimental contexts. This notion is supported by evidence showing that reductions in CSE are associated with lower pain severity (Chowdhury et al. 2022). Indeed, suppressed CSE may subserve higher-order strategies in response to sustained painful input (see, for example, Legrain et al. 2011).

Across two experiments, this study replicated previous findings that TMS-evoked MEPs in the FDI muscle are inhibited by a preceding electrical stimulus to the ipsilateral index finger (Tokimura et al. 2000; Helmich et al. 2005; Tamburin et al. 2005; Bikmullina et al. 2009; Tamè et al. 2015). Specifically, early intervals (ie 25 to 35 ms) produced inhibition, followed by recovery at 45 ms, facilitation at 60 ms, and returning inhibition at 160 ms. The time-dependent modulation of CSE likely reflects sensorimotor interactions that facilitate hand–object interactions (Johansson and Flanagan 2009). The facilitation observed at 60 ms aligns with previous reports (Tamburin et al. 2001; Roy and Gorassini 2008; Devanne et al. 2009) and we propose may reflect activation of the long-latency reflex (LLR) pathway, which follows the H-reflex 50 to 100 ms following limb displacement or submaximal nerve stimulation (Pruszynski and Scott 2012).

We also showed that this pattern of AI was consistent across tactile stimulus durations of 0.2 and 0.4 ms. The absence of a significant difference between these pulse widths, despite our initial hypothesis, suggests that AI may not scale linearly with incremental increases in afferent recruitment by modest increases in pulse width. Although longer pulse duration can activate a larger population of afferent fibers (Chipchase et al. 2011), the 0.2-ms increment used may not have been sufficient to generate additional volleys capable of producing measurable changes in CSE. Relatedly, it is possible that AI reflects a threshold-based mechanism rather than a proportional relationship between afferent drive and inhibition, such that additional afferent input will not necessarily amplify inhibitory effects. It is also plausible that AI is more sensitive to changes in perceived stimulus intensity than to changes in pulse duration alone (Bailey et al. 2016), meaning that manipulations not altering the subjective intensity may not translate to stronger physiological modulation. Future work may benefit from exploration of a wider range of afferent durations and intensities to identify the boundary conditions under which tactile input modulates AI.

When interpreting the findings of the current study, it is important to recognize that MEP amplitudes alone cannot distinguish between spinal and supraspinal processes. While LAI is suggested to be cortically driven, as its timing exceeds that of spinal reflexes (Chen et al. 1999), the origins of SAI remain less certain (Turco et al. 2018). Nonetheless, early studies using H-reflex responses and transcranial electrical stimulation showed that pain-related inhibition of CSE is primarily cortical (Valeriani et al. 1999, 2001; Farina et al. 2001) or a combination of cortical and later H-reflex modulation (Le Pera et al. 2001). This underscores the need for further research to clarify the interaction between spinal and supraspinal processes in shaping functional motor responses to both noxious and innocuous stimuli. In the present study, CSE, SAI, and LAI were assessed during pain. However, previous studies show that SAI and LAI can change after pain ceases (Burns et al. 2016b; Delahunty et al. 2024). Future studies should therefore assess these phenomena postpain to better understand sensorimotor-related plasticity after exposure to and recovery from noxious input.

## Conclusion

The present study demonstrated that moderate tonic heat pain applied to the left forearm reduced TMS-evoked MEP amplitudes in the left FDI muscle, while tactile AI remained unaffected. These findings indicate that tonic nociceptive input suppresses CSE, while concurrent transient tactile–motor interactions are preserved. Importantly, not only did pain fail to alter AI, but also such tactile inputs leading to AI did not modulate the overall suppressive effect of pain on CSE. This suggests that pain-related reductions in motor excitability and tactile afferent inhibition can operate in parallel without influencing one another. While pain-induced CSE inhibition aligns with existing literature, our findings extend this by showing that forearm pain can alter motor activity in the distal hand. More broadly, these findings, observed in healthy participants, have clinical relevance for understanding how pain can selectively affect motor processes while the other networks of sensorimotor interactions remain unaffected.

## Supplementary Material

Supplementary_Materials_bhag041

## Data Availability

Raw data are openly available on OSF (https://osf.io/nhkm4/).
