# Supplementary material for: Pain suppresses corticospinal excitability, independent of tactile afferent inhibition
Source: Cereb Cortex. 2026 Apr 16;36(4):bhag041. doi: 10.1093/cercor/bhag041 (PMC13089548; doi:10.1093/cercor/bhag041)
Supplement: Supplementary_Materials_bhag041 [file supplementary_materials_bhag041.docx]

Supplementary Materials

Pain suppresses corticospinal excitability, independent of tactile afferent inhibition

Louisa Gwynne^*^ and Luigi Tamè^*^

School of Psychology, University of Kent, Canterbury, UK

*Address for correspondence:

Louisa Gwynne or Luigi Tamè

E-mail: lg497@kent.ac.uk; l.tame@kent.ac.uk

School of Psychology

University of Kent

CT2 7NP, Canterbury, United Kingdom

**S1**

**M1 localisation and TMS threshold intensity estimation**

A 3x3 grid with 10 mm spacing was drawn onto the participant’s reconfigured digitalised scalp in BrainSight, centred on our lab average right M1-FDI (at rest) coordinates from previous experiments or, in the case of Experiment 1 Session 2, the grid was centred on the participant’s right M1-FDI MNI co-ordinates determined in Experiment 1 Session 1. Starting at the central grid point, TMS was applied at 35% of the maximum stimulator output, increasing in increments of 5% until muscle evoked activity was visible on the EMG recording. The coil was moved across the grid, stimulating each grid point twice to find the optimal location (i.e. maximal MEP output); the intensity was adjusted in increments of 5% as appropriate. Next, a new 3x3 grid with 5 mm spacing was centred on this optimal location and the procedure was repeated. Finally, coil orientation was adjusted until desired MEPs were recorded. On average, the coil handle remained 45 degrees relative to the sagittal plane. The mean average right M1-FDI MNI coordinates were 35.0, -5.1, 58.8 (x,y,z).

**S2**

**Electrocutaneous stimulation and thresholding**

Innocuous transcutaneous electrical stimulation was controlled in MATLAB (version 2019b) through a National Instruments data-acquisition card (6001). Square-wave pulse widths were either 0.4 ms or 0.2 ms; session 1 and 2, respectively). This method generated a singular, fast onset-offset tap-like sensation to the index finger by momentary activation of predominantly Aβ mechanoreceptive fibres. The participants’ electrotactile sensory detection threshold (SDT; the minimum stimulator output detectable by the participant) was estimated using a 2-down/1-up automated staircase procedure. This converges to an approximate 71% perceivability threshold (Levitt, 1971). Starting at 0.1 mA, the stimulation increased by 0.3 mA step-sizes, reduced to 0.1 mA after the first reversal and to 0.02 mA after the second reversal. The staircase ended at the eighth reversal and the SDT was calculated as the average of the last two reversals. During the main afferent inhibition experimental protocol, electrotactile stimulation was delivered at 2.5 times the SDT; all participants reported this as perceivable but not salient or painful.

References

Levitt, H. (1971). Transformed up-down methods in psychoacoustics. *The Journal of the Acoustical Society of America*, *49*(2B), 467–477.
